# Evaluation of the Therapeutic Effect of the Traditional Herbal Medicine *Atrifil* and *Oshagh* Gum on Testosterone-Induced Benign Prostatic Hyperplasia in Wistar Rats

**DOI:** 10.1155/2022/5742431

**Published:** 2022-07-05

**Authors:** Fatemeh Akbari, Mohammad Azadbakht, Anand Gaurav, Fatemeh Azimi, Zahra Mahdizadeh, Lale Vahedi, Ayob Barzegar Nejad, Aroona Chabra, Mohammad Eghbali

**Affiliations:** ^1^Department of Pharmacognosy, Faculty of Pharmacy, Mazandaran University of Medical Sciences, Sari, Iran; ^2^Faculty of Pharmaceutical Sciences, UCSI University, Kuala Lumpur, Malaysia; ^3^Faculty of Pharmacy, Mazandaran University of Medical Sciences, Sari, Iran; ^4^Department of Pathology, Faculty of Medicine, Mazandaran University of Medical Sciences, Sari, Iran; ^5^Department of Urology, Faculty of Medicine, Mazandaran University of Medical Sciences, Sari, Iran; ^6^Pharmaceutical Sciences Research Center, Faculty of Pharmacy, Mazandaran University of Medical Sciences, Sari, Iran

## Abstract

Benign prostatic hyperplasia (BPH) is a common disease that affects elderly men with various complications. This study evaluates the effects of an Iranian traditional herbal medicine “Atrifil and Oshagh gum” on BPH in male Wistar rats. *Atrifil* is a combination of three medicinal plants: *Emblica officinalis Gaertn, Terminalia chebula Retz, and Terminalia bellerica Retz*” extracts, and *Oshagh* gum is *Dorema ammoniacum* D. Dono gum. In this study, 30 male Wistar rats were divided into five groups: normal control, disease, finasteride, and extract with 300 and 600 mg/kg groups. The extract is a combination of hydroalcoholic *Atrifil* extract and *Oshagh* gum. All groups received intramuscular testosterone enanthate to induce BPH except the normal control group. On the twenty-eighth day, prostate glands were separated. Histopathological changes were observed. Furthermore, the prostate-specific antigen (PSA) and prostate weights were measured. The binding propensities of finasteride, equol, and flavonoids present in this extract such as quercetin, rutin, and kaempferol for 5*α*-reductase, estrogen receptor alpha and beta, and estrogen-related receptor gamma were assessed using in silico docking approach. Histopathological evaluation, biochemical parameter, and PSA level results indicated significant inhibition of accruing and progression of BPH in groups treated with 600 mg/kg extract (*p* < 0.01). Furthermore, molecular docking showed that rutin had a high affinity to bind the receptors 5*α*-reductase, estrogen receptor beta, and estrogen-related receptor gamma even more than finasteride, and on average, quercetin had a higher affinity to all these receptors. In the end, it can be concluded that *Atrifil* and *Oshagh* gum is effective in preventing BPH.

## 1. Introduction

Benign prostatic hyperplasia (BPH) is a chronic andrological disease, which has a high prevalence among men. Its prevalence is approximately 60% in people aged 50 to 60, and it increases to up to 70% in men in the sixth decade of life. BPH, the nonmalignant growth of prostate glands, is associated with urinary obstruction in men [1–5]. This can affect the quality of life, and patients may suffer from complicated problems such as urinary retention, frequent urination, urinary tract infection, and bladder stones [6–12]. Many factors are involved, which can lead to this disease such as imbalance in sexual hormones, especially androgens and their metabolites, aging, and dietary lifestyle.

Researchers reported that lifestyle is also an important factor in BPH; for instance, daily aerobic exercise can reduce insulin, insulin-like growth factor I, and estradiol/testosterone ratio level in plasma, particularly when coupled with a low-fat and high-fibre regimen consisting of whole grains, fruits, and vegetables [13]. Moreover, they verified the relation between BPH and high blood pressure, obesity, and metabolic diseases such as diabetes [14–16].

Androgen is not directly correlated with BPH, but excessive androgen hormone can be observed in this phenomenon [17, 18]. Androgens are converted to estrogen and dihydrotestosterone with aromatase enzyme and 5*α*-reductase, respectively. The activation of these enzymes increases with age. Due to the decrease in testosterone levels and the increase in its metabolites and the role of estrogen on prostate cell proliferation, the risk of getting BPH increases [19, 20].

The mechanism of dihydrotestosterone is that it binds itself to androgen receptors and signals the transcription of mitogenic factor of the epithelial and stromal cells [21]. This incident occurs in combination with the effect of both of the given hormonal factors [19, 22].

Aging, fibrosis, and weakening of prostate muscle tissue play a key role in BPH. The fluid produced by the prostate gland accumulates in the gland because of the weakening of the prostate muscle. This accumulation leads to tissue damage, and the tissue filled the damaged area with collagen fibre. Collagen fibre replacement in tissue reduces normal tissue function. Dysfunction of the gland leads to more fluid accumulation. Taken together, the abnormal cycle of progressive muscle tissue fibrosis and fluid accumulation is the main agent causing benign prostatic hyperplasia [23, 24].

*Atrifil* extract and *Oshagh* gum (AO) are a combination of three medicinal fruits: *Emblica officinalis Gaertn* (from Phyllanthaceae family), *Terminalia chebula Retz* (from Combretaceae family), and Terminalia bellerica *Retz* (from Combretaceae family) and the gum of *Dorema ammoniacum* D. Don (from Apiaceae family). *Emblica officinalis* (*E. officinalis*) is a rich source of vitamin C, flavonoids, tannins, terpenoids, and alkaloids, which cause various biological activities [25, 26]. *E. officinalis* extract has antioxidant activity against alcohol-induced oxidative damage in rat liver microsomes [27]. It helps thymocytes against arsenic-induced apoptosis in mice with anti-apoptosis function [28]. *E. officinalis* extract has anti-inflammatory effect in rodent models in the condition of both acute inflammation and chronic inflammation [29]. Also, *E. officinalis* has antidiabetic effect in type 2 diabetes [30], and it showed antihypertensive effect in deoxycorticosterone acetate salt-induced hypertension in animal model [31] and showed efficacy in anticancer in cervical cancer cells [31, 32].

*Terminalia bellerica Retz* is a plant from Combretaceae family, and the fruit is ovoid in shape and grey to dark brown in color. It contains fixed oil along with a phenolic ester known as hexahydroxydiphenic acid ester and also has triterpenoids such as arjungenin and bellericagenin A and B in *Terminalia bellerica Retz* fruit [33, 34].

It has antioxidant and antimicrobial effects because of its polyphenol combination and has analgesic activation effects thanks to its TNF-*α* inhibition [35]. *Atrifil* prevents DNA destruction by radicals so it has an anticancer effect. It is proved that *Atrifil* can reduce lipid levels in the blood in order of HMG-COA enzyme inhibition [36].

*Terminalia chebula Retz* contains several components such as tannins, flavonoids, sterols, amino acids, fructose, resin, fixed oils, and also high phenolic content [37], and its fruits have high amount of different pyrogallol (hydrolyzable) type tannins (up to 32% tannin content) such as gallic acid, chebulic acid, punicalagin, chebulanin, corilagin, neochebulinic acid, ellagic acid, and chebulagic acid [38, 39]. Other phytochemicals such as anthraquinones, ethanedioic acid, sennoside, 4,2,4 chebulyl-d-glucopyranose, terpinenes, and terpineols have also been reported to exist in this plant [40, 41]. Furthermore, triterpenoids and their glycosides have been isolated from *T. chebula* stem bark [42]. The ethnobotanical uses of the fruit of this plant are for dysuria and retention of urine, and it is also useful in renal calculi [43]. Studies showed indicated that *Terminalia chebula* extract has a suppressive effect on the growth of prostate cancer cells by androgen receptor-mediated transcription regulation [44, 45].

*Dorema ammoniacum D. Don* is native to Central and Eastern Iran [42]. The plant contains several components such as free salicylic acid, ammoresinol, doremin, doremine A, ammodoremin, and oleo gum resin [46–48]. The gum is used in Iranian traditional medicine and also in Indian traditional medicine for treating several complications such as respiratory tract diseases, expectorant, stimulant and antispasmodic, gastrointestinal discomfort, and urinary tract problems [49–52].

Therefore, the combinations of these three extracts with the highest phenol and flavonoid contents as mentioned in Iranian traditional medicine have a great potential to treat complications [52]. Despite many efforts to find a solution for these phenomena, cost-benefit management for the treatment of BPH is still an unsolved problem. The aim of this study was the evaluation of the combination of the *Atrifil* extract and *Oshagh* gum: an Iranian traditional medicine with a high content of phenolic and flavonoid components as antioxidant for preventing BPH that contributed to oxidative stress.

## 2. Materials and Methods

### 2.1. Preparation of Hydroalcoholic *Atrifil* Extract and *Oshagh* Gum

The fruits of *Emblica officinalis Gaertn, Terminalia bellerica Retz*, and *Terminalia chebula Retz* and the gum of *Dorema ammoniacum D. Don* were bought from a grocery store in Sari, Mazandaran, Iran, and approved by a systematic herbalist. They were dried in a dryer and ground to sieve with mesh of 80. 250 g ground part of fruits of *Emblica officinalis Gaertn, Terminalia bellerica Retz*, and *Terminalia chebula Retz* was extracted by maceration with 70% ethanol at room temperature for 3 days, and the extracts were dried under vacuum by rotary evaporator followed by freeze drier. Then, it was kept in a tight container and put in a refrigerator. Three grams of each extract and 3 grams of the gum were combined and dissolved in the lowest amount of normal saline for homogenization, and this combination was named “AO.”

### 2.2. Flavonoids and Total Phenolic Content Determination

The aluminum chloride colorimetric method was used for the measurement of flavonoids [53], and the Folin-Ciocalteu assay method was used to measure the total phenolic content of each plant, respectively, and a spectrophotometer instrument was used for reading the absorbance [54, 55].

### 2.3. Liquid Chromatography Mass Spectrometry

The *Atrifil* extract and *Oshagh* gum LC/MS was conducted according to the Herbani method [56]. For this purpose, an Agilent series 6100 LC/MS system (Agilent Technologies, Santa Clara, CA, USA) with a photodiode array detector was set at 257 nm. A 150 mm × 3.0 mm, 3.5 *μ*m Waters XTerra MS with symmetry C_18_ (5 *μ*m, 20 × 3.9 mm) was used. The mobile phase was consisted of (1) water with 0.1% (v/v) formic acid and (2) acetonitrile with 0.1% (v/v) formic acid using gradient solvent system of 10–90% (v/v) (2) for 26 minutes. The temperature was 35°C, and flow rate was 0.3 ml/min.

The mass spectrometer is directly joined to the LC system without stream splitting and by using an electrospray interface Model HP 59987A. The nebulizer pressure, temperature of drying gas (N2), and gas flow rate were 5.5 × 10^5^ Pa, 300°C, and 40 ml/min, respectively.

### 2.4. Animals and Experimental Design

Eight-week-old male Wistar rats (200 to 250 g) were purchased from the Institute for Laboratory Animal Research of Mazandaran University of Medical Science and kept under standard laboratory conditions (half-day light/dark cycle at 22 ± 3°C). Animal rights were respected, and the study procedure was in accordance with the Association for the Protection of Animal Rights with Ethical Code IR principles (MAZUMS.REC.1398.2740).

30 male rats were divided into five groups, each containing six rats in every cage, group 1 (normal group) did not receive anything, group 2 (disease group) was treated with just 25 mg/kg of intramuscular testosterone enanthate in corn oil, group 3 (standard medicine group) was treated with 25 mg/kg of IM injection of TE and received 10 mg/kg of finasteride by gavage, and groups 4 and 5 were treated with 25 mg/kg of IM injection of TE and, respectively, received 300 mg/kg and 600 mg/kg AO extract by gavage once a day during 28 days (the dose of testosterone enanthate and finasteride was selected based on relevant articles, with consideration of the weight of the animals [57]).

### 2.5. Body Weight and Prostate Weight

Initial body weights and the final weights and prostate weights were measured after the animals were anesthetized by i.p injection of ketamine-xylazine mixture with a dose of 0.15 ml/100 mg per body weight at the end of the experiment. The ratio of prostate weight to body weight was calculated as prostate index as follows:

(1) $$\begin{array}{c} \text{Prostate index}=\frac{\text{prostate weight}}{\text{body weights}}. \end{array}$$

### 2.6. Parameter Evaluated

At the end of the experiment, the prostate gland was collected and half of the group's prostate glands were analyzed for oxidative stress biochemical parameters including malondialdehyde (MDA), glutathione (GSH), and protein carbonyl groups.

### 2.7. Malondialdehyde (MDA)

Malondialdehyde (MDA) was measured according to the Satoh method [58, 59]. The reaction of samples with thiobarbituric acid (from Merck Company) in the presence of normal butanol was the base of MDA measurement. The spectrophotometric method was used for measuring the absorbance of the sample at 532 nm and compared it with the standard curve. Butanol was used as blank [59, 60].

### 2.8. Glutathione (GSH)

Glutathione (GSH) measurement was conducted by homogenizing the tissue and separating the supernatant and then mixing with Tris buffer 0.02% containing 0.02 EDTA (pH = 8.9) and 0.01 molar DTNB (5,5′-dithiobis-(2-nitrobenzoic acid)). The absorbance was measured at 412 nm according to the Sedlak and Lindsay method [61, 62].

### 2.9. Protein Carbonyl Groups

Protein carbonyl groups were measured based on Levine et al.'s method [63]. The hydrazine is induced by the reaction between 2 and 4-dinitrophenylhydrazine (DNPH) and carbonyls. The hydrazine absorbance was measured by a spectrophotometry at 405 nm [64].

### 2.10. PSA Content

At the end of the experiment, blood samples were collected under anesthesia directly from the heart for the evaluation of free prostate-specific antigen (PSA) with the PSA ELISA Kit [65].

### 2.11. Histopathological Examination

10% formalin was used for fixing the prostate tissue. To investigate morphologic changes, prostate samples were sectioned at a 4 *μ*m thickness and stained with hematoxylin and eosin (H&E) for histological evaluation under a 40x microscopic light. The changes in stroma tissue size, epithelial cell, and inflammatory of the prostate tissue were investigated. For each change, 0 to 3 scores were considered in comparison with the normal group (Table 1) [66–68].

### 2.12. Protein Preparation

The crystal structures of 5AR, ER*α*, ER*β,* and ERR*ɣ* with PDB ID of 7BW1, 1R5K, 1X7B, and 2GPV, respectively were retrieved from the Protein Data Bank (https://www.rcsb.org).

The enzyme was saved into dockable Protein Data Bank, partial charge, and atom type (PDBQT) format in preparation for molecular docking.

### 2.13. Ligand Preparation

SDF structures of finasteride (CID 57363), equol (CID 91469), kaempferol (CID 5280863), quercetin (CID 5280343), and rutin (CID 5280805) were retrieved from the PubChem database (https://www.pubchem.ncbi.nlm.nih.gov). The compounds were converted to the dockable PDBQT format using AutoDock tools.

### 2.14. Molecular Docking

Docking of finasteride, equol, and flavonoids present in this extract such as quercetin, rutin, and kaempferol for 5*α*-reductase, ER*α*, ER*β*, ad ERR*ɣ* was assessed using in silico docking approach. For this purpose, PyRx.lnk software was used and binding affinities were determined [69].

The enzymes and ligands were dragged into their respective columns in their PDBQT form, and the software was launched. The binding affinities of the ligands and enzymes were recorded. Molecular interactions between the compounds and for 5*α*-reductase, estrogen receptor alpha and beta, and estrogen-related receptor gamma were viewed with BIOVIA Discovery Studio 2020 software. The binding profiles were determined using Discovery Studio Visualizer.

### 2.15. Statistical Analysis

Data from the results were expressed as mean ± standard error of the mean (SEM). Evaluation between group results was carried out with the one-way ANOVA test followed by Tukey's test, and the statistically significant value of *p* was considered to be <0.05. Statistical analysis was performed with GraphPad Prism Software (GraphPad Software Inc., USA).

## 3. Results

### 3.1. Phenol and Flavonoid Contents of the Extracts

The flavonoid amount in the extracts of the *Terminalia chebula Retz*, *Terminalia bellerica Retz,* and *Emblica officinalis Gaertn* was calculated using the standard curve equation of quercetin (*y* = 0.0069*x* + 0.0347, *r*^2^ = 0.9967) and expressed as quercetin equivalents, and *Dorema ammoniacum D. Don*, *Terminalia chebula Retz*, *Terminalia bellirica Retz,* and *Emblica officinalis Gaertn* were measured as 18/24 ± 0/968, 158/8 ± 13/241, 173/287±/159, and 269/16 ±6/285 (mg/1 g of dry extracts), respectively.

In addition, the phenol content was measured using the Folin-Ciocalteu assay method with the standard curve equation (*y* = 0.0061*x* + 0.0682, *r*^2^ = 0.9992) and expressed as gallic acid equivalents. *Dorema ammoniacum D. Don*, *Terminalia chebula Retz*, *Terminalia bellerica Retz,* and *Emblica officinalis Gaertn* were measured as 13.086 ± 1.845, 141/4 ± 92/176, 169/75 3±/218, and 257/28 ± 2/129 (mg/1 g of dry extracts), respectively.

### 3.2. LC-MS Analysis of *Atrifil Extract and Oshagh* Gum

**Figure 1:**
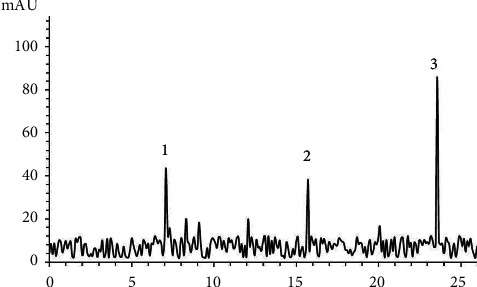
LC-MS analysis of the *Atrifil* extract and *Oshagh* gum. 3 flavonoids were detected by LC-MS.

LC-MS analysis has been done to determine the active compounds of *Atrifil* extract and *Oshagh* gum. The result had identified 3 active compounds of the flavonoid group namely quercetin, routine, and kaempferol (Table 2 and Figure 1).

### 3.3. Prostate Weight and Index

**Figure 2:**
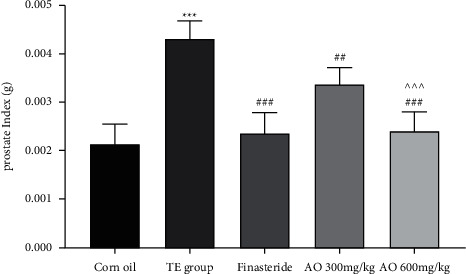
Prostate index of groups; normal control (corn oil), disease (testosterone group), finasteride, and extract with 300 and 600 mg/kg (AO) groups. Group 1 (normal group), group 2 (disease group), group 3 (standard medicine group), and groups 4 and 5 treated with 300 mg/kg and 600 mg/kg AO extract for 28 days. The values are expressed as mean ± SEM (*N* = 6 animals/group). ^*∗*^−*p* < 0.05, ^*∗∗*^−*p* < 0.01, and ^*∗∗∗*^−*p* < 0.001 vs. control, and ^#^*p* < 0.05, ^##^*p* < 0.01, and ^###^*p* < 0.001 vs. TE group.

The prostates weights of the groups were compared with the weight of the normal group on the final day of the study. Testosterone enanthate (25 mg/kg) administration increased prostate index and weight in rats significantly (*p* < 0.001) compared with the normal group. The finasteride group compared with the disease group showed a significant decrease in prostate weight (*p* < 0.001) (Figure 2). The samples treated with AO (300 mg/kg and 600 mg/kg) (<0.01 and *p* < 0.001, respectively) prostate weight decreased dose-dependently compared with the testosterone group.

### 3.4. Biochemical Parameter Evaluation

**Figure 3:**
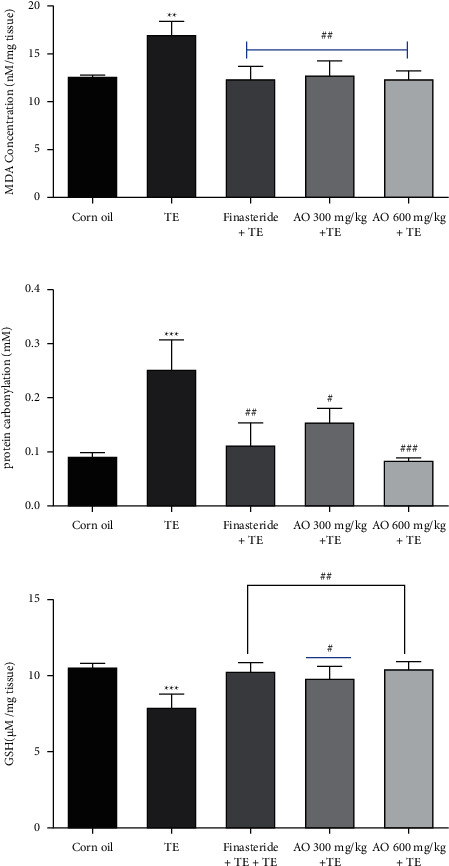
GSH, MDA, and protein carbonyl level of prostate gland tissue, and the values are expressed as mean ± SEM (*n* = 3 animals/group). ^###^*p* < 0.001, ^##^*p* < 0.01, and ^#^*p* < 0.05 vs. testosterone, ^*∗∗∗*^*p* < 0.001 and ^*∗∗*^*p* < 0.01 vs. control.

According to Figure 3, the results showed a decrease in GSH (*p* < 0.001) level in the TE group in comparison with the control group; however, AO extract with doses of 300 mg/kg and 600 mg/kg showed increases in GSH level (respectively, *p* < 0.01, *p* < 0.05, *p* < 0.01) as same as the finasteride group.

The protein carbonyl group results showed increases in the TE group (*p* < 0.001) and decreases in finasteride and AO extract with doses of 300 and 600 mg/kg (respectively, *p* < 0.05, *p* < 0.05, *p* < 0.01) compared with the TE group.

The MDA result indicated a significant increase in MDA level as the lipid peroxidation index in the TE group (*p* < 0.001) and decrease in groups finasteride and AO 300 and 600 mg/kg (respectively, *p* < 0.01, *p* < 0.01, *p* < 0.01).

### 3.5. PSA

**Figure 4:**
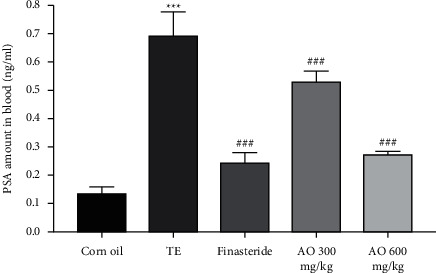
PSA level on the 28th day of experiment; normal control (corn oil), disease (testosterone group), finasteride, and extract with 300 and 600 mg/kg (AO) groups. AO extracts were significantly different from the disease group (*p* < 0.05).

As seen in the testosterone group, PSA level was elevated (*p* < 0.001) compared with the normal group. The results indicated a meaningful relationship between decreasing PSA content and treatment with AO extracts (300 and 600 mg/kg) compared with the disease group (*p* < 0.001). The finasteride group showed a similar effect. No significant difference between finasteride and AO extract with dose of 600 mg/kg was observed (Figure 4).

### 3.6. Histopathological Examination

**Figure 5:**
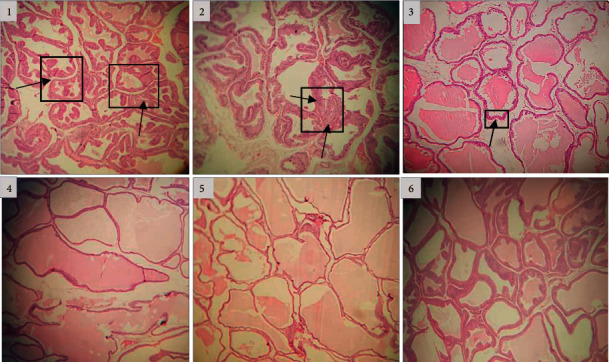
Microscopic images (40x) of the prostate tissue (H&E). The arrows and squares represent morphology changes such as stromal proliferation, abnormal acinar fold, and hypertrophy, ((1) and (2)) treatment with muscle injection testosterone, (3) treatment with extract at dose of 300 mg/kg, (4) treatment with extract at dose of 600 mg/kg, (5) treated with finasteride as standard medicine, and (6) treated with corn oil.

According to Figure 5, the disease group indicated an obvious disruption in prostatic tissue shown as inflammatory cell hyperplasia, hypertrophy of the epithelium, and progression of stromal proliferation. These results are significant when *p* < 0.001. A meaningful reduction in the combination effect (300 mg/kg) on the progression of stromal proliferation, inflammatory cell hyperplasia, and epithelial cell hypertrophy was found compared with the disease group (Table 3).

A clear benefit of finasteride in the prevention of inflammatory cell hyperplasia, epithelial cell hypertrophy, and progression of stromal proliferation was identified in this test (*p* < 0.01, *p* < 0.001, *p* < 0.01, respectively).

### 3.7. Docking Result

Estrogens play an important role in BPH. Estrogen-related receptor gamma (ERR-*γ*) decreases the rate of proliferation and slows the progression rate of prostate cancer [63–65]. ER*α* and ER*β* (activation transcription factor ligands) mediated the estrogen effect. ER*α* mediates bladder enlargement in male mice treated with testosterone and 17*β*-estradiol. Estrogen and dihydrotestosterone (DTH) are metabolite of 5*α*-reductase effect on endogenous androgens [20].

**Figure 6:**
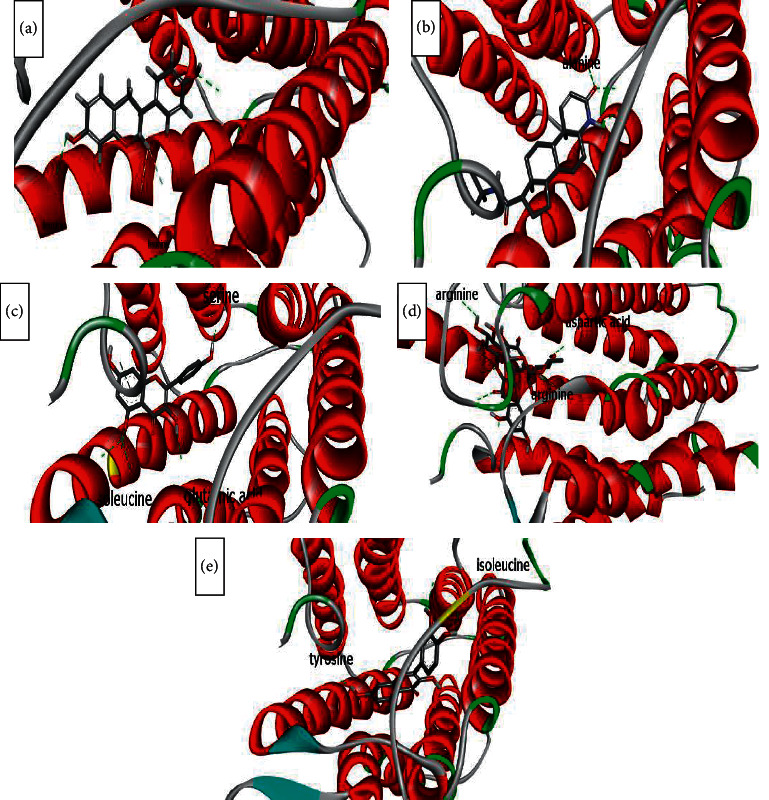
Interaction between amino acids in the binding site of 5*α*-reductase enzyme, and (a) = equol, (b) = finasteride, (c) = kaempferol, (d) = quercetin, and (e) = rutin, respectively. The green bonds show intramolecular hydrogen bonds. Arginine, aspartic acid, alanine, glutamic acid, tyrosine, isoleucine, alanine, and serine are involved in H-bond interaction between ligands and receptors.

**Figure 7:**
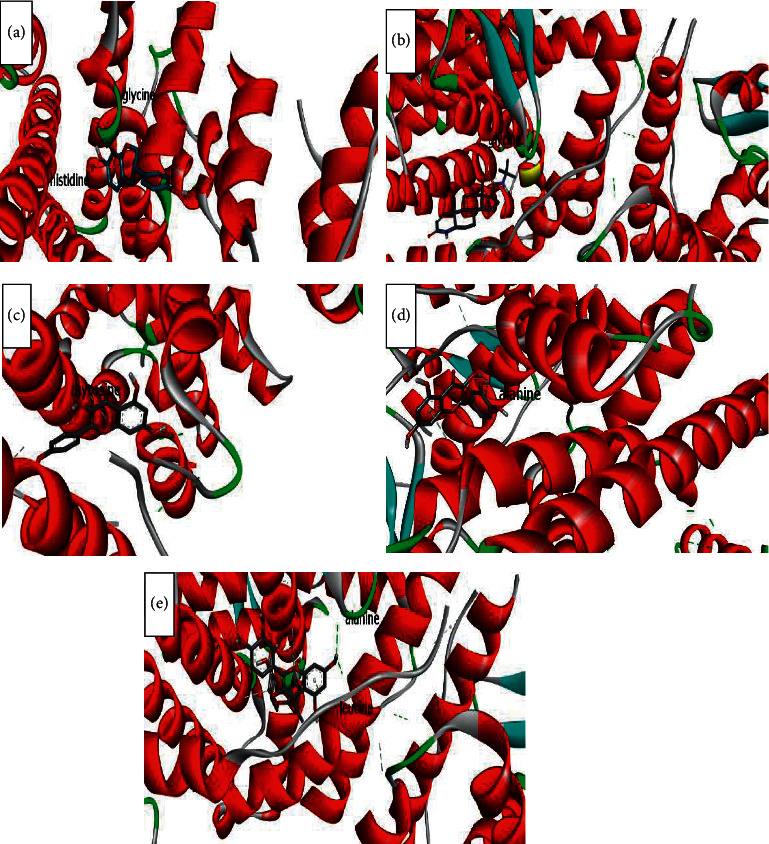
Interaction between amino acids in the binding site of ER-a enzyme, and (a) = equol, (b) = finasteride, (c) = kaempferol, (d) = quercetin, and (e) = rutin, respectively. The green bonds show intramolecular hydrogen bonds. Glycine, histidine, tyrosine, alanine, and leucine from ER-a enzyme are involved in H-bond interaction with ligands.

**Figure 8:**
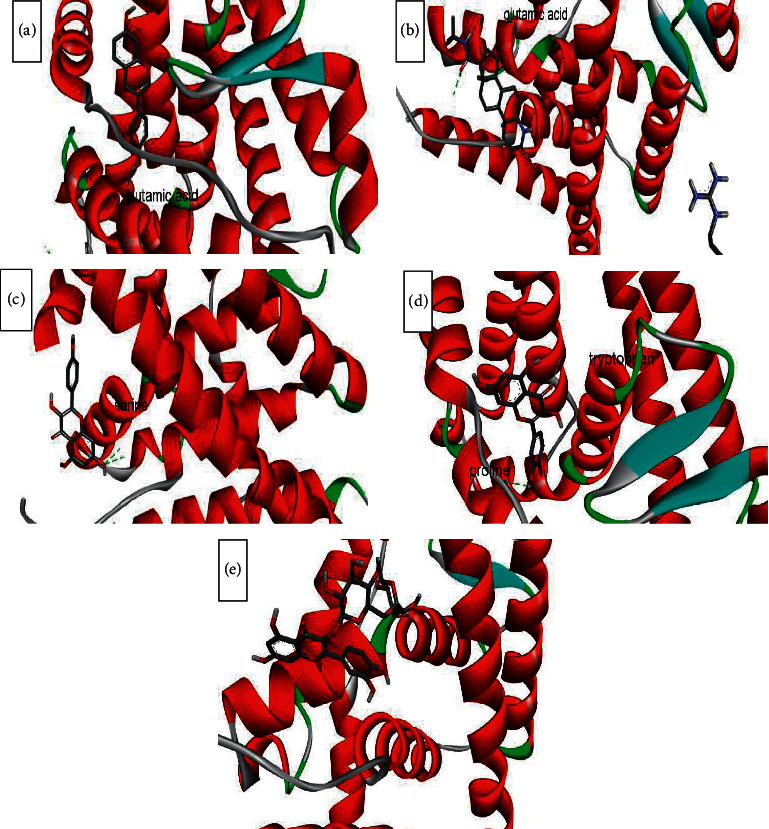
Interaction between amino acids in the binding site of ER-b enzyme, and (a) = equol, (b) = finasteride, (c) = kaempferol, (d) = quercetin, and (e) = rutin, respectively. The green bonds show intermolecular hydrogen bonds. Glutamic acid, serine, tryptophan, and proline from ER-b enzyme are involved in H-bond interaction with ligands.

**Figure 9:**
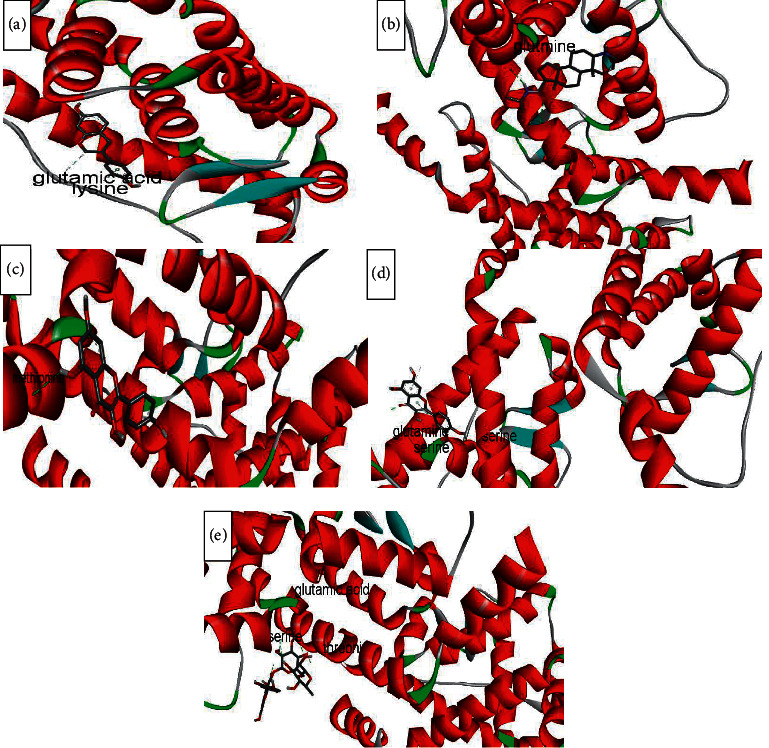
Interaction between amino acids in the binding site of ER-gamma enzyme, and (a) = equol, (b) = finasteride, (c) = kaempferol, (d) = quercetin, and (e) = rutin, respectively. The green bonds show intermolecular hydrogen bonds. Glutamic acid, glutamine, serine, methionine, and threonine from ER-gamma enzyme are involved in H-bond interaction with ligands.

The binding affinity to receptors (estrogen-related receptor gamma, ER-b, ER-a, 5*α*-reductase) is represented in Table 4. The minimum binding energy is related to the highest binding affinity. In silico study (Table 4) showed that rutin binding energy (releasing energy when it binds to the receptor) was −10.6 Kcal/mol to 5*α*-reductase enzyme compared with −9.2 Kcal/mol exhibited by finasteride as presented in Figures 6–9, but rutin needs +13.4 kcal/mol energy to bind to the ER-a receptor.

Amino acids involved in H-bond interaction between 5*α*-reductase and ligands are arginine, aspartic acid, alanine, glutamic acid, tyrosine, isoleucine, alanine, and serine. Glutamic acid, glutamine, serine, methionine, and threonine from ER-gamma enzyme are involved in H-bond interaction with ligands. Furthermore, glycine, histidine, tyrosine, alanine, and leucine from ER-a and glutamic acid, serine, tryptophan, and proline from ER-b enzyme are involved in H-bond interaction with ligands.

ER*α* ligand-binding domain (LBD) structure consists of 11 *α*-helices. The attachment to this enzyme includes hydrogen bonds for the steroid/hormone ligand and hydrophobic interactions for the nonsteroidal ligand. Hydroxyl groups of estradiol as in positions 3 and 17 of the A and D rings have hydrogen bonded to Glu353, Arg394, and a water molecule and His524. ER*β* can bond to estradiol and genistein with hydrogen bonds of hydroxyl moieties with receptor histidine.

Studies showed that if a special substance tends to affect Er*α* and Er*β* as an agonist or relative agonist it should be placed at the interaction distance of the active site of amino acids Glu353, Arg394, and His524, but for an antagonist, it differs and just one missing interaction can have the opposite effect [70]. To evaluate the physiological behavior of ligands in the vicinity of enzymes, the binding affinity (binding energy) of ligands to receptors is important (Figures 6–9).

## 4. Discussion

BPH is a chronic andrological disease, in which inflammation is an important stage of it. Frequent inflammation induced by testosterone in the prostatic epithelial cells over time leads to hyperplasia in these cells. The result of this hyperplasia is urinary retention, frequent urination, urinary tract infection, and bladder stones [71].

This study investigated the efficacy of *Atrifil* extract and *Oshagh* gum. By administering muscle injection of TE (25 mg/kg) for 28 days, prostatic hyperplasia was induced in this period, which was observed in Akbari et al.'s study as well [10].

Enzymes are involved directly in these diseases or increasing the progression of the disease such as 5*α*-reductase, estrogen-related receptor gamma (ERR-*γ*), and estrogen receptors *α* and *β*. Estrogen has an important role in the progression, growth, and proliferation of prostate cells and, with combination of DTH, can have an extra effect on BPH [20]. Estrogen-related receptor gamma (ERR-*γ*) and ER*β* increase positive estrogen-like influences in the prostate, and ERR-*γ* has been shown to slow proliferation in prostate and breast cancer cell lines [72–74]. ER*α* and ER*β* (activation transcription factor ligands) mediated the estrogen effect. Exogenous estrogens affected the prostatic epithelial, urothelial, and bladder fibroblast with the mediation of ER*α* and make proliferation. Studies showed that ER*α* mediates bladder enlargement in male mice treated with testosterone and 17*β*-estradiol, but this effect has not been observed in ER*β* [75].

Estrogen and dihydrotestosterone (DTH) are metabolite of 5*α*-reductase effect on endogenous androgens [20]. Finasteride reduces the level of DTH in blood by reducing the activity of 5*α*-reductase and decreases the oxidative stress induced by DHT and preserves the body's antioxidants [76].

In vitro examination indicated that EER-*ɣ* decreases the rate of proliferation and slows the progression rate of prostate cancer [72–74]. Equol is a flavonoid known as an antioxidant with anti-inflammatory effects, and it showed activity against BPH by increasing the transcriptional activity of EER-*ɣ* and anti-androgenic effect or selective androgen modulator activities [77–81].

In this study, it was observed that kaempferol, quercetin, and rutin have higher affinity to binding to 5*α*-reductase and equol, and quercetin and rutin have higher affinity to ER*β* in comparison with finasteride. Furthermore, these ligands do not have the required qualification to be agonists (binding to Glu353, Arg394, and His524 amino acids for ER*α* receptor and hydrogen bonds to histidine for ER*β*), and due to their high binding affinity, they potentially have antagonistic effects.

Numerous studies have been conducted on BPH in the condition of in vivo during 4 weeks, which is similar to this study [82–84]. In their study, Sik Shin et al. evaluated Yukmijihwang-tang efficacy in the treatment of BPH in Wistar rats. Testosterone propionate was daily treated on rats to induce the disease [82]. Testosterone enanthate is faster-acting than testosterone propionate, and this form of the testosterone (testosterone enanthate) was utilized in this study. Curcumin is well-known agent for anticancer therapy, and many studies evaluated this compound on testosterone-induced BPH. After castration of rats, the procedure of the treatment with subcutaneous testosterone was conducted. Curcumin inhibited BPH progression [85].

Finasteride as standard treatment of BPH was used. Studies used 5 and 10 mg/kg of finasteride as the standard medicine [85–88]. In this study, we used 10 mg/kg finasteride.

*Atrifil* extract *and Oshagh* gum are a combination of tree medicinal fruits: *Terminalia chebula Retz* (from Combretaceae family) and *Terminalia bellerica Retz* (from Combretaceae family) and *Emblica officinalis Gaertn* (from Phyllanthaceae family) and *Dorema ammoniacum D. Don* (from Apiaceae family) gum. Oshagh (the gum of *Emblica officinalis Gaertn*) is an Iranian traditional medicine, and the gum is commonly used for many complications such as respiratory tract diseases, expectorant, stimulant, and antispasmodic and urinary tract problems [49–51].

In this study, we observed that the AO, which is rich in phenolic and flavonoid compounds, the known profile of phenolic and flavonoid compounds of this combination formula, is gallic acid, ellagic acid, methyl gallate, quercetin, rutin, and kaempferol according to literature studies [89–92]. Finasteride and equol were used as the standard substance to compare the binding affinity of the quercetin, rutin, and kaempferol. Equol has a flavonoid-like structure and shows significant affinity to bind ER*β* and ER*α,* and also, 5*α*-reductase and finasteride are a 5*α*-reductase inhibitor. Therefore, this study investigated the effects of this extract based on traditional medicine and the affinity for receptor binding.

In this study, biomarker examination showed a significant decrease in PSA level, prostate index, and the progression of prostatic hyperplasia in extract groups in comparison with the disease group. Furthermore, no significant correlation was found between 600 mg/kg extract and finasteride. This finding shows that the efficacy of the extract with a dose of 600 mg/kg was the same as the finasteride treatment of BPH. Many studies indicated that *Terminalia chebula Retz* has anti-inflammatory and anti-cell proliferation effects and contributed this effect to the phenolic compound of this extract [35, 37, 93]. *Terminalia bellerica Retz* has also shown anti-cell proliferation effect [94, 95]. *Dorema ammoniacum D. Don* gum showed to have anti-inflammatory effects by inhibiting anti-inflammatory mediator release in animal studies [96, 97]. BPH is related to inflammation, cell proliferation, and oxidative stress. [98–100] The antioxidants are free radical scavengers that inhibit the cell damage and related disease. In this study, the administration of testosterone induces oxidative stress in prostate tissue and it is observed in the disease group. It is evidenced by an increased level of PCO and MDA and lowered levels of GSH in the disease group. Furthermore, this biomarker was near to normal range in group treated with 300 and especially 600 mg/kg AO extract.

Due to the high amount of flavonoids and phenolic compounds represented in the extract, and the antioxidant capacity of these compounds, this antioxidant capacity can be the possible mechanism of this extract in dealing with BPH.

The purpose of this study was to determine the effect of the combination of AO formula on BPH. One of the more significant findings to emerge from this study is that AO extract can reduce BPH complications by inhibiting cell division and anti-inflammatory effect. In addition, flavonoid of this extract such as kaempferol, quercetin, and rutin showed high affinity to 5*α*-reductase, ERR-*γ*, ER*α,* and ER*β* even more than finasteride, especially 5*α*-reductase enzyme.

## 5. Conclusions

AO extract inhibited testosterone-induced hyperplasia in rats. Due to inflammation's role in the BPH, this inhibition was probably related to the regulation of inflammatory responses by reducing oxidative stress based on the high amount of phenolic and flavonoid compounds. It should be mentioned that due to some limitations, intracellular events, and cell signaling procedures, it was not done. It would suggest evaluating each tissue enzyme (5*α*-reductase, ER*α* and ER*β*, EER-*ɣ*) expression in the presence of AO extract in the prostate ligand.

## Figures and Tables

**Table 1:** Histopathological characteristic aspect.

Character	Score			
Stromal tissue size	0 (normal)	1 (slight increase)	2 (significant increase)	3 (intense increase)
Epithelia cell size	0 (normal)	1 (slight increase)	2 (significant increase)	3 (intense increase)
Inflammatory	0 (not seen)	1 (few number)	2 (Significant number)	3 (large number)

**Table 2:** LC-MS chromatogram of *Atrifil* extract and *Oshagh* gum.

Peak IDs	Retention time (min)	Molecular weight	Concentration (*μ*g/g)
(1) Rutin	7.09	608.50	65.49085
(2) Kaempferol	15.73	284.50	57.51008
(3) Quercetin	23.07	300.50	128.8624

**Table 3:** Histopathological characteristic aspect.

Histopathological changes	Score				
Groups	Normal group	Disease group	Extract 300 mg/kg	Extract 600 mg/kg	Standard medicine group
Epithelia cell size	68/0 ± 16/2^*∗∗∗*^	74/0 ± 33/2	0 ± 0.0^###^	0 ± 0.0^###^	68/0 ± 16/2^*∗∗∗*^
Inflammatory	0/0 ± 0	37/0 ± 83/1^*∗∗∗*^	0 ± 0.0^#^	0 ± 0.0^#^	0 ± 0.0^##^
Stromal tissue size	0/0 ± 0	50/0 ± 5/2^*∗∗∗*^	37/0 ± 16/1^#^	57/0 ± 00/1^##^	57/0 ± 00/1^##^

**Table 4:** Binding affinity of the finasteride, equol, kaempferol, quercetin, and rutin to 5*α*-reductase, estrogen receptor alpha and beta, and estrogen-related receptor gamma.

Substance	5*α*-reductase (kcal/mol)	ER-a (kcal/mol)	ER-b (kcal/mol)	Estrogen-related receptor gamma (kcal/mol)
Finasteride	−9.2	−6.2	−7.1	−6.9
Equol	−9.1	−5.9	−7.5	−7.2
Kaempferol	−9.2	−2.7	−6.2	−5.8
Quercetin	−9.6	−6.9	−7.7	−6.6
Rutin	−10.6	+13.4	−7.7	−7.4

## Data Availability

All materials including rats, testosterone enanthate, PSA ELISA, Kit, finasteride, and ethanol for extraction are provided by Mazandaran University of Medical Science, and data are obtained from the Laboratory of Mazandaran University of Medical Science.
